# Circulating Biomarkers of Diabetic Retinopathy: An Overview Based on Physiopathology

**DOI:** 10.1155/2016/5263798

**Published:** 2016-06-08

**Authors:** Olga Simó-Servat, Rafael Simó, Cristina Hernández

**Affiliations:** ^1^Diabetes and Metabolism Research Unit, Vall d'Hebron Research Institute, Universitat Autònoma de Barcelona, Barcelona, Spain; ^2^Centro de Investigación Biomédica en Red de Diabetes y Enfermedades Metabólicas Asociadas (CIBERDEM), Instituto de Salud Carlos III (ISCIII), Barcelona, Spain

## Abstract

Diabetic retinopathy (DR) is the main cause of working-age adult-onset blindness. The currently available treatments for DR are applicable only at advanced stages of the disease and are associated with significant adverse effects. In early stages of DR the only therapeutic strategy that physicians can offer is a tight control of the risk factors for DR. Therefore, new pharmacological treatments for these early stages of the disease are required. In order to develop therapeutic strategies for early stages of DR new diagnostic tools are urgently needed. In this regard, circulating biomarkers could be useful to detect early disease, to identify those diabetic patients most prone to progressive worsening who ought to be followed up more often and who could obtain the most benefit from these therapies, and to monitor the effectiveness of new drugs for DR before more advanced DR stages have been reached. Research of biomarkers for DR has been mainly based on the pathogenic mechanism involved in the development of DR (i.e., AGEs, oxidative stress, endothelial dysfunction, inflammation, and proangiogenic factors). This review focuses on circulating biomarkers at both early and advanced stages that could be relevant for the prediction or detection of DR.

## 1. Introduction

Diabetic retinopathy (DR) is the most frequent complication of diabetes and the main cause of blindness in working-age adults in the developed countries [1]. DR prevalence in the diabetic population is around one-third, with one-tenth having vision-threatening states such as diabetic macular edema (DME) or proliferative diabetic retinopathy (PDR) [2]. Moreover DR entails considerable costs related to both treatment and social support [3, 4]. As the disease remains asymptomatic until the pathology is significantly advanced, screening to detect it during the early stages is necessary [5].

The actual available treatments for DR are applicable only at advanced stages of the disease and are associated with significant adverse effects [6–8]. In early stages the only therapeutic strategies that physicians can offer are a tight control of the risk factors for DR. The principal risk factors for developing DR are hypertension, glycemic control, and diabetes duration [9–20]. However, clinical studies in diabetic patients reveal a substantial variation in the onset and severity of DR [21–24], thus indicating that genetic factors may influence the susceptibility to developing DR [25].

In order to develop new therapeutic strategies for early stages of DR new diagnostic tools are urgently needed. In this regard, circulating biomarkers could be useful (i) to detect early disease, (ii) to identify diabetic patients most prone to progressive worsening, in whom intensified therapy could be prioritized, and (iii) to monitor the effectiveness of new drugs for DR before advanced DR stages have developed.

A biomarker has been defined as “a biological molecule found in blood, or other bodily fluids, or tissue which represents a sign of a normal or abnormal process of a condition or disease.” Therefore, “a biomarker may be used to see how well the body responds to a treatment for a disease or condition” [26, 27]. Biomarkers may help to identify people with subclinical disease and also to monitor the clinical disease [28], for example, to assess treatment response. Ideally, a biomarker has to be measured in accessible tissues [28]. As the retina constitutes a small proportion of total body weight, a circulating biomarker for DR should be highly specific to the retina rather than a marker of systemic vascular disease.

Research of biomarkers for DR has been based on the pathogenic mechanism involved in the development of DR. In this review we will summarize the more important molecules that could become biomarkers for DR.

## 2. Advanced Glycation End Products

The nonenzymatic glycation reaction is known to be one of the most significant mechanisms contributing to tissue damage seen in diabetes. It involves a complex series of chemical reactions that lead to the formation of early glycation products, alpha-dicarbonyls, which are directly toxic to both tissues and precursors of AGEs (advanced glycation end products). AGE accumulation contributes to diabetic complications through direct tissue damage as well as through the activation of specific AGE receptors (RAGE) [29–32].

Several AGEs have been proposed as biomarkers for DR. N-Epsilon-carboxymethyl lysine (N-*ε*-CML), the most abundant of circulating AGEs, has been extensively studied. N-*ε*-CML has been found elevated in the serum of diabetic patients and to an even higher extent in those with microvascular complications [33–37]. Interestingly, Choudhuri et al. [38] found that subjects with nonproliferative diabetic retinopathy (NPDR) showed a significantly higher level of N-*ε*-CML compared to the PDR group. Secondly, pentosidine is an AGE that has also been related to DR, and some studies found its blood levels elevated in patients with PDR compared with NPDR or patients without DR [35, 36, 39]. However, in the EURODIAB study [40] the association between pentosidine and DR disappeared after controlling for diabetes duration. As in the case of N-*ε*-CML, Salman et al. [41] detected a significant elevation of pentosidine levels in patients during the earliest detectable phase of DR (early NPDR) and more elevation at the preproliferative stage, returning to lower levels at the proliferative stage of DR. These findings suggest that both pentosidine and N-*ε*-CML can be used as a biochemical marker for the early occurrence of DR and as a warning factor in the preproliferative stage of DR. More prospective studies are needed to confirm these findings, with the glomerular filtration rate (GFR) being taken into account, because AGEs tend to increase with the reduction of the GFR [36]. Additionally, skin collagen pentosidine and N-*ε*-CML levels, measured in human skin punch-biopsies samples, also predicted the progression of DR in two prospective studies [42, 43].

Highly reactive dicarbonyl compounds such as 3-deoxyglucosone (3-DG) have been identified as important intermediates of the glycation reaction, not only as precursors of AGEs but also for their direct effects in cell functions [44, 45]. Kusunoki et al. [46] found that 3-deoxyglucosone levels were higher in diabetic patients than in control subjects and further increased according to the severity of the DR.

As previously mentioned, some of the nonenzymatic glycation consequences are due to AGEs binding their receptor (RAGE). Soluble RAGE blood levels are low in patients with diabetic complications in comparison with diabetic patients without complications and nondiabetic subjects [37]. By contrast, increased levels of this receptor in patients with PDR compared with those with NPDR or without DR have also been reported [47, 48].

RAGEs activation induces both permeability of microvascular endothelial cells and production of reactive oxygen species (ROS) [49, 50]. Choudhuri et al. [38] found that ROS in peripheral blood mononuclear cell (PBMC ROS) increased significantly in NPDR and PDR subjects compared to diabetic patients without DR and control subjects. This increase strongly correlated with N-*ε*-CML. Some authors have proposed N-*ε*-CML as the key molecule for triggering the production of ROS, leading, for example, to lipid peroxidation and oxidative DNA damage [51]. Malondialdehyde (MDA), which is a metabolite produced during phospholipid peroxidation, is increased in subjects affected with PDR compared to those affected with NPDR or healthy controls [51, 52], further suggesting that oxidative stress and lipid peroxidation are involved in the DR progression. Further data is required to establish the role of soluble RAGE, PBMC ROS, and MDA as possible biomarkers of DR.

## 3. Biomarkers of Basement Membrane and Extracellular Matrix Turnover

DR is associated with important disturbances in the structure and metabolism of basement membranes [53–55]. Basement membrane thickening and increased vascular permeability are two major retinal vascular changes associated with the pathogenesis of DR [56–58]. Collagen IV and laminin are components of the basement membranes [59] and have been proposed as biomarkers of DR.

Collagen IV is a major matrix protein of the basement membranes, and elevated serum and urine levels have been associated with diabetic microangiopathic complications, especially DN [60–64]. Lee et al. [59] found that plasma levels of 7S-collagen, a collagen IV domain resistant to various proteases, are elevated in DR and DN and that its levels increased with the severity of both diseases. The authors also found that diabetic patients without complications had higher levels of this domain than healthy subjects. Kotajima et al. [65] also found higher levels in serum and also in the vitreous fluid of diabetic patients with DR than in those without this disease. However there are few prospective studies evaluating the role of collagen IV as a DR biomarker and most of them had a relatively small number of subjects.

Laminin is a noncollagenous glycoprotein of basement membranes which is upregulated by high glucose concentrations [66, 67]. It has been postulated that serum levels of this protein, or its fragments, could reflect the changes observed in the basement membranes of diabetic patients. Although the association between laminin-P1 concentrations, the largest pepsin resistant fragment of laminin, and DN seems established [68–72], the results concerning DR are less clear. In this regard, whereas Pietschmann et al. [68] and Hayashi et al. [60] found no correlation between DR and laminin-P1, our group has established a positive correlation between laminin-P1 and DR in a diabetic population [73]. In addition, we demonstrated that panretinal photocoagulation significantly reduced the increased serum Lam-P1 levels detected in diabetic patients [74]. This was certainly intriguing, given that retinal microcirculation represents only a minor part of the total number of blood vessels in the body. Moreover, our findings suggest that the contribution of the Lam-P1 produced in the retina to its circulating levels is significant and that serum Lam-P1 levels could be a useful biomarker for DR. In a prospective study we also found that circulating laminin-P1 was not a useful predictor of DR development (at least over a 4-year period) but that it was an early marker of the presence of DR, as well as a marker of its severity [75].

Metalloproteinases (MMPs) are a family of zinc-dependent proteinases that degrade extracellular matrix proteins and are modulated by endogenous tissue inhibitors of metalloproteinases (TIMPs). In DR the balance between MMPs and TIMPs is impaired and increased levels of MMP-9 and MMP-2 are found in the retina and vitreous. MMPs appear to play a multiple role, being proapoptotic, proinflammatory, and proangiogenic, and MMP-9 seems to act as a modulator of inflammation in the pathophysiology of DR [76, 77]. Furthermore, increased MMPs may be implicated in the disruption of the blood retinal barrier (BRB), an early event in the development of DR [77].

MMP-9 is the largest and the most complex member of the MMP family and TIMP-1 shows a greater preference for MMP-9 [77]. Jacqueminet et al. [78] found that type 1 diabetic patients have both higher circulating levels of MMP-9 and a higher MMP-9/TIMP-1 ratio. In the same study, patients affected with DR showed elevated systemic MMP-9 and an elevated MMP-9/TIMP-1 ratio compared to patients without DR. Similarly, Béranek et al. [79] found both MMP-2 and MMP-9 elevated in plasma of PDR patients [80]. Recently, in the EURODIAB Prospective Complication Study, based on a cohort of type 1 diabetic subjects, plasma MMP-2 levels showed a significant positive association with the development of PDR at 6–9 years of follow-up. It should be emphasized that this study was prospective and that the analyses were adjusted for cardiovascular risk factors and HbA1c [81].

## 4. Biomarkers Related to Inflammation

A large body of evidence supports the role of inflammation mediators in the pathogenesis of DR [82–86]. Some of the molecules implicated in both systemic and local inflammation have been tested as possible biomarkers of DR. The EURODIAB Prospective Complication Study hypothesized that a *Z*-score composed by C-reactive protein (CRP), Tumor Necrosis Factor-*α* (TNF-*α*), and interleukin-6 (IL-6) could be associated with the presence of vascular complications in diabetic patients. They found a positive correlation between these inflammatory factors and DR, DN and cardiovascular disease (CVD) [40]. In this section, we will focus on the possible role of these inflammatory molecules as biomarkers of DR.

CRP belongs to the pentraxin family of calcium-dependent ligand-binding proteins and is produced in the liver in response to IL-6. It is an acute-phase protein and a marker of inflammation and tissue damage [87]. CRP has been associated with macrovascular disease and with DN [88, 89]. Some studies have found a positive association between CRP levels and the prevalence of DR, in both type 1 and type 2 diabetic patients [90–93]. By contrast, other authors have found no relationship between CRP and DR [94–98] or with its progression [99]. However, these conflicting results could be due to some confounding factors that were not taken into account. For example, in the EURODIAB study [40], the association between CRP and DR decreased after adjustment for the body mass index (BMI). In fact, the BMI is intimately related to CRP levels. Lim et al. [100] in a population-based, cross-sectional study of 718 persons with diabetes in the Singapore Malay Eye Study (SiMES) reported that diabetic patients with higher levels of CRP and a higher BMI were less likely to have DR. The authors propose that this protective effect could be due to its proangiogenic properties, which may be beneficial in the preproliferative stages of DR, and also due to its anti-inflammatory effects. Further studies would help determine the possible protective effect of CRP in DR.

IL-6 has been found elevated in the vitreous fluid of diabetic patients with DR and has been implicated in the pathogenesis of DR [101, 102]. Higher levels of systemic IL-6 were detected in diabetic children with DR than in those without DR [103]. Moreover, Shimizu et al. [104] found that serum IL-6 concentration correlates significantly with the severity of macular edema and could be a predictor of PDR. However, these results have not been confirmed in larger studies [95, 96, 98].

TNF-*α* is a cytokine that promotes the irreversible adhesion of leukocytes to the endothelium (leukostasis), increases the production of reactive oxygen species, and is implicated in BRB breakdown [105, 106]. A strong correlation between plasma levels of TNF-*α* and PDR has been reported [98, 107]. Klein et al. [97] reported that this correlation was mediated by the presence of kidney disease. In children, Zorena et al. [108] found that the risk of NPDR was strongly dependent on TNF-*α* levels. Finally, it has been reported that baseline circulating TNF-*α* is a predictor of DR incidence [109] as well as of the progression of diabetic complications [110]. Interestingly, it has been observed that the TNF-*α* level in tears is highly correlated with DR severity [111].

### 4.1. Adhesion Molecules

The adhesion molecules are implicated in leukostasis and some of them act as angiogenic factors. Leukostasis plays an important role in diabetic vascular leakage, capillary nonperfusion, and endothelial cell damage [85]. Adhesion molecules are also markers of endothelial dysfunction [112]. The most important are intercellular adhesion molecule-1 (ICAM-1), vascular cell adhesion molecule-1 (VCAM-1), and E-selectin, and these are found in high concentrations in the vitreous of patients affected with PDR [113–116]. Matsumoto et al. [117] found that ICAM-1 was associated with the presence of DR, but not with macroangiopathy, while E-selectin and VCAM-1 were associated with both micro- and macroangiopathy complications. VCAM-1 has been associated with the presence of DR [97, 118, 119], and both VCAM-1 and E-selectin have been associated with the presence of DR, DN, and CVD [120]. On the other hand, in the Hoorn study [121], a *Z*-score combining CRP and ICAM was found to be associated with the presence of DR. Moreover, Roy et al. [109] found in a prospective study in an African-American population that baseline ICAM-1 levels were associated with the incidence of DME and that baseline E-selectin levels were associated with DR progression. In addition, Spijkerman et al. [99] in a prospective study found that baseline E-selectin levels were also associated with DR progression, whereas VCAM-1 was not significantly associated with the presence or progression of DR. However, none of these findings have been supported by other studies [98, 122–124]. Notably, Uğurlu et al. [125] assessed the levels of ICAM-1 and VCAM-1 in early stages of DR (when a biomarker is much needed) and found no differences between patients with or without DR.

## 5. Miscellaneous Candidate Proteins

### 5.1. Retinol-Binding Protein 4

Retinol-binding protein 4 (RBP4) is a transport protein for vitamin A and also an adipokine, which is secreted by hepatocytes and adipose tissue [126]. Graham et al. [127] found that serum levels of RBP4 are related to insulin resistance in subjects with obesity, impaired glucose tolerance, or T2DM. Moreover, elevated levels are associated with increases in the BMI, the waist-to-hip ratio, triglyceride levels, and systolic blood pressure and with decreased high-density lipoprotein cholesterol levels; all of them are components of the metabolic syndrome. As the levels of RBP4 are inversely correlated with the expression of GLUT4 in adipocytes, it has been postulated that elevated RBP4 contributes to insulin resistance downregulating GLUT4 [128].

Inflammation plays an essential role in DR development [82, 83]. In this regard, RBP4 has been correlated not only with obesity and insulin resistance but also with inflammatory factors such as C-reactive protein and IL-6 [129]. It has been demonstrated that RBP4 induces inflammation in human retinal endothelial cells by stimulating the expression of proinflammatory molecules [130]. Moreover, in an animal model overexpressing RBP4, early-onset microglia activation followed by progressive retinal degeneration mediated by an increased expression of pro-IL-18 was observed [131]. Therefore, RBP4 could be involved in the inflammatory process of DR and could be considered a biomarker of the early stages of DR. Takebayashi et al. [132] found elevated RBP4 levels in T2DM patients in comparison with nondiabetic subjects and significantly increased levels in patients with PDR versus no DR or nonproliferative DR. However, this relation was reduced after adjusting for urinary albumin excretion (UAE). Moreover, Li et al. [133] found that both UAE and serum RBP4 levels were significantly higher in PDR patients. Nevertheless, other studies have not found any association between RBP4 levels and DR [134, 135].

There are some factors that could influence RBP4 levels such as the BMI, some drugs (i.e., antidiabetic and hypolipidemic agents), vitamin A deficiency, and the GFR [126, 132, 134]. Therefore, further research taking into account these confounding factors is needed.

### 5.2. Adrenomedullin

Adrenomedullin (ADM) is a peptide that was first isolated from the acid extract of human pheochromocytoma [136]. Later, it was found to act as a circulating hormone [137] produced mainly in the vascular endothelium rather than in the adrenal medulla [138]. ADM has various functions including vasodilatation, the regulation of vascular cell growth, hormone secretion, and natriuresis [139]. In the eye ADM is produced by the retinal pigment epithelium (RPE), vascular endothelial cells, fibroblasts, macrophages, hyalocytes, and glial cells [140]. ADM has been related with the pathophysiology of many vitreoretinal disorders and inflammatory retinal diseases and its plasma levels have been found to be higher in patients with diabetes [141]. In addition, elevated levels of ADM have been reported in the vitreous fluid from patients with DR [142, 143], as well as in retinal membranes of PDR patients [143]. Notably vitreous ADM correlated with the prognosis of this disease [143].

Elevated plasma levels of ADM are found in patients with DR compared to controls and diabetic patients without DR [144, 145]. However, in a group of children and adolescents with T1DM without funduscopic alteration but with functional abnormalities in the ERG examination, ADM levels were not increased [146]. These findings suggest that circulating levels of ADM are not sensitive enough to detect neurodegeneration and that more advanced stages of DR are needed to induce a significant increase in ADMA plasma levels.

### 5.3. Homocysteine

Homocysteine is a sulfur-containing amino acid formed during the metabolism of methionine [147, 148]. This molecule is considered to be a risk factor for cardiovascular disease, although the exact mechanism by which homocysteine causes atherosclerosis is little known. Experimental evidence suggests that homocysteine produces an endothelial dysfunction through ROS, decreases the production of endothelial-derived nitric oxide, stimulates vascular smooth-muscle cell proliferation, increases the formation of highly atherogenic oxycholesterols and lipid peroxidation, has a thrombogenic effect [147], and has a stimulatory effect on the expression of VEGF [149].

Although some studies, mostly performed in type 2 diabetic patients, have established an association between plasma levels of homocysteine and DR [148, 150, 151], especially with PDR [149, 152–155] or DME [156, 157], these findings have not been confirmed in other studies [158, 159]. Moreover, in a recent study [149] an association between plasma homocysteine and vitreous homocysteine in PDR was found, the increased vitreous levels being attributed to BRB breakdown. Further experimental studies are needed to establish the role of homocysteine in the pathogenesis of DR.

The level of homocysteine is higher in males and increases with age, renal impairment, the use of certain drugs (i.e., metformin, phenytoin, and methotrexate), and the nutritional deficiency of vitamin cofactors (i.e., folate, vitamin B12, and vitamin B6) required for homocysteine metabolism [148, 149, 160]. Therefore, these factors should be taken into account when the relationship between homocysteine levels and the presence of DR is being analyzed. In this regard, case-control studies adjusted for the major risk factors for DR and for determinants of homocysteine concentrations, including vitamin B12 and folate levels, have shown a positive association between homocysteine and DR [150, 160]. As mentioned above, renal disease is a potential confounding factor. Pepys and Hirschfield [87] found an association between homocysteine and vision-threatening DR, but this was no longer significant after adjustment for serum creatinine and the UAC (urine albumin creatinine) ratio. However, in other studies the association persisted after adjusting for renal function [150, 156, 157, 160].

In conclusion, homocysteine could be a biomarker of DR, but most of the studies have demonstrated a relationship in advanced stages of the disease. Further studies taking into account possible confounding factors such as renal function are required.

### 5.4. ADMA

Endothelial dysfunction and impaired ocular hemodynamics underlying DR development are associated with decreased nitric oxide (NO) synthase activity and NO bioavailability, thus resulting in vasoconstriction and increased ROS. Serum asymmetric dimethylarginine (ADMA) is involved in the NO pathway and serum levels of ADMA have been found elevated in diabetic patients with DR [161–163].

The exact mechanism leading to the elevation of the ADMA level has not been clarified, but decreased activity of dimethylarginine dimethylaminohydrolase (DDAH), which metabolizes ADMA to citrulline and dimethylamine, has been implicated because DDAH is inactivated by ROS [164, 165]. Notably, ADMA has a NO synthase-independent action that upregulates ACE (angiotensin-converting enzyme) expression and also promotes vasoconstriction and vascular thickening in eNOS knock-out mice [166].

## 6. Endothelial Progenitor Cells (EPCs)

EPCs are marrow-derived cells involved in adult neovascularization and endothelial homeostasis and are known to be stimulated by several modulators such as VEGF, erythropoietin, and substance P [167–169]. EPCs are increased in PDR [170–173], especially in mature forms [173]. On the other hand, some authors found a decreased level of EPCs in NPDR [172, 173], but these findings were not confirmed in other studies [171].

It has been postulated that low levels of EPCs in peripheral blood contribute to macrovascular diabetic complications [174, 175] while an increase of these levels is related to DR. Fadini et al. [170] called this phenomenon the “diabetic paradox” and suggested that an increase of growth factors and/or cytokines contributes to preferential homing of EPCs to the retina rather than to diabetic hearts or limbs. In this regard, Zerbini et al. [176] found that one of the EPCs, the CFU-Hill cells, which are positive for both endothelial markers and hematopoietic and monocytic lineage markers, manifests abnormalities in association with the presence of NPDR and possibly before clinical evidence of retinopathy. These abnormalities result in a reduced expression of adhesive molecules, which may have consequences for facilitating the progression of retinopathy. Moreover, Tan et al. [177] found that EPCs were impaired in their ability to migrate and to repair damaged capillary endothelium in PDR patients.

## 7. Immunocomplexes and Autoantibodies

It has been postulated that humoral factors have a possible role in the pathogenesis of vascular complications [178, 179]. Indeed, immunocomplexes (IC) and anti-cardiolipin antibodies of the IgM type are found in higher levels in diabetic patients with vascular complications [179]. However, few studies have specifically addressed their potential role as a biomarker of DR.

In diabetes, damage to the BRB enables extravasation and subsequent lipoprotein modification, which are sufficient to initiate the synthesis of autoantibodies that, in turn, lead to the formation of IC. These IC in DR have been associated with pericyte loss, leukostasis, the activation of macrophages, and the stimulation of growth factors. Lopes-Virella et al. [180, 181] reported the association of IC containing advanced glycation end product- (AGE-) LDL (AGE-LDL) and oxidized LDL (oxLDL) with the presence and progression of DR.

On the other hand, some antibodies have been also associated with DR. Li et al. [182] in* in vitro* studies found that retinal pericyte-reactive antibodies induced cellular damage by activating complement. In addition, anti-pericyte antibodies have been found in T2D patients especially during earlier stages of DR, probably due to a reaction with antigens expressed by “activated” pericytes [183, 184]. In advanced stages of DR the prevalence of antibodies declines and this could mark pericyte loss and disease progression [184]. Moreover, antibodies against CD38 (antigen that is present on retinal pericytes) have been found in the serum of diabetic patients [185–188]. Further studies are needed to elucidate the role of these antibodies as biomarkers in DR.

## 8. Circulating RNA

Nucleic acids have been identified in peripheral blood, so providing a new potential tool for diagnosis and/or prognosis. It has been suggested that the concentration of plasma nucleic acids (DNA and RNA) reflects the degree of cell death. In fact, they are increased in numerous pathological processes, for example, in numerous cancers [189]. We will review the implications of circulating mRNA and microRNA as possible biomarkers for DR.

### 8.1. mRNA

Hamaoui et al. [190] investigated mRNA levels of rhodopsin in plasma samples from diabetic patients with different degrees of DR. Rhodopsin is a visual pigment found exclusively in the rod cells of the retina [191] and it was detected in peripheral blood of healthy individuals and diabetic patients with and without DR. With the exception of PDR, there was a trend for the levels of mRNA of rhodopsin to increase with the severity of retinopathy. In the case of the PDR group in which there were no differences with the group of healthy individuals, the authors argued that in this state the cells are exhausted in a metabolic capacity or in total number [190]. In a further study they combined the detection of mRNA from rhodopsin with mRNA from retinal amine oxidase, a protein also exclusively expressed in the retina which is found to decrease with the progression of DR, and calculated the ratio between them. The ratio had a higher area under the ROC curve than rhodopsin alone, which allowed them to differentiate mild from severe DR [192].

Other potential biomarkers are mRNA circulating levels of RPE65 and retinoschisin, both exclusive of the retina. Shalchi et al. have demonstrated that circulating RPE65 mRNA increases with DR severity while plasma mRNA levels from retinoschisin decrease [193].

The authors of these studies suggest that the possible mechanisms by which circulating mRNA levels of RPE65 and retinoschisin increase with the severity of DR are release from dead and dying retinal cells and possible upregulation of their transcription and/or controlled secretion of their mRNA [190, 193]. However, it should be noted that in all of these cases the mRNA was also detected in healthy and diabetic patients without retinopathy. Thus, longitudinal studies could help explore the role as predictors of DR development.

### 8.2. MicroRNA

MicroRNAs (miRNAs) are endogenously produced short coding RNAs of about 20–22 nucleotides that have an important role in modulating gene expression, inhibiting the expression of their target genes via posttranscriptional mechanisms [194–196]. Due to their stability in biofluids such as blood and urine, because it is relatively easy to quantify them, and because some of them are cell-type or tissue-specific, miRNAs are potential biomarkers for the early detection of DR [197, 198]. In recent years, several miRNAs have emerged as important regulators of particular aspects of DR pathology [199]. Indeed, there are some miRNAs which have been found decreased in animal models of DR, such as miRNA-146a, miRNA-200b, and miRNA-29b, whose target genes are fibronectin (implicated in fibrosis and basement membrane thickening), VEGF (an angiogenic factor), and PAX (an activator of a proapoptotic pathway), respectively. The reduction of these miRNAs is related to overexpression of the corresponding proteins in DR [200–202]. Recently, García de la Torre et al. [203] performed a study in a group of type 1 diabetic patients measuring the expression of miR-126 in EPCs and found increased levels in those patients with DR.

However, although the tide of miRNA research is rising fast, the potential for miRNAs to act as noninvasive biomarkers and even therapeutic targets has yet to be elucidated.

## 9. Concluding Remarks

In order to develop therapeutic strategies for the early stages of DR new diagnostic tools are urgently needed. In this regard, circulating biomarkers could be useful not only for detecting those patients at early stages of the disease but also for identifying those patients most prone to progressive worsening. In this subset of patients the tight control of blood glucose levels and blood pressure should be prioritized.

A limiting factor in searching for biomarkers of DR is that their plasma changes can reflect systemic effects of long-standing diabetes rather than specific damage in the retina. In this regard, the studies addressed to investigate circulating levels of proteins and/or mRNAs that are exclusively expressed in the retina should be prioritized. The combination of specific markers of DR and general markers of diabetes-induced microangiopathy would appear as feasible means of advance in this research area. On the other hand, the identification of protective biomarkers is a challenge still to be met. The comparison by means of high-throughput methods of plasma samples from patients with long-standing diabetes without DR with those patients with DR could help us in identifying these “protective” proteins or metabolites.

Finally, circulating biomarkers could be useful not only for identifying the early stages of DR but also for monitoring the effectiveness of new drugs and for identifying the group of patients whose response is the most significant. Such a strategy would permit us to optimize the resources of healthcare system, but this will require the combined efforts of ophthalmologists, diabetologists, basic researches, and healthcare providers.
